# Multiparametric Ultrasound for Preoperative Assessment of Parotid Tumors: A Novel Diagnostic Pathway

**DOI:** 10.1002/hed.70036

**Published:** 2025-09-06

**Authors:** Katharina Margherita Wakonig, Markus Herbert Lerchbaumer, Philipp Arens, Heidi Olze, Steffen Dommerich, Thomas Fischer

**Affiliations:** ^1^ Department of Otorhinolaryngology Charité – Universitätsmedizin Berlin, Corporate Member of Freie Universität Berlin, Humboldt‐Universität Zu Berlin, and Berlin Institute of Health Berlin Germany; ^2^ Department of Radiology Charité ‐ Universitätsmedizin Berlin, Corporate Member of Freie Universität Berlin, Humboldt‐Universität Zu Berlin, and Berlin Institute of Health Berlin Germany

**Keywords:** contrast‐enhanced ultrasound, diagnostic accuracy, multiparametric ultrasound, parotid gland tumors, real‐time imaging, shear wave elastography

## Abstract

**Background:**

Accurate preoperative differentiation of parotid gland tumors (PGTs) is essential for facial nerve preservation. This study evaluates a novel, real‐time multiparametric ultrasound (mpUS) approach combining B‐mode, shear wave elastography (SWE), and contrast‐enhanced ultrasound (CEUS), based on qualitative image interpretation.

**Methods:**

Eighty‐nine patients with 91 PGTs underwent mpUS prior to surgery or ultrasound‐guided biopsy. Two blinded experts independently reviewed the images and reached consensus through real‐time discussion. Only qualitative features were assessed. Histopathology served as the reference.

**Results:**

mpUS achieved 83% sensitivity (95% CI: 62.6–94.0), 94% specificity (95% CI: 84.3–98.1), and 91% accuracy (95% CI: 82.4–96.3). CEUS and SWE provided complementary perfusion and stiffness data. CCDS was excluded due to limited value.

**Conclusion:**

mpUS improves diagnostic accuracy compared to single‐modality US and supports early, targeted treatment. Limitations include the small number of malignancies, single‐center design, and qualitative evaluation. Broader use may require training and equipment access.

## 1 Introduction

The parotid gland is divided into a superficial and deep lobe by the peripheral part of the facial nerve [1]. Most parotid gland tumors (PGTs) arise in the superficial lobe. While the majority of PGTs are benign, with pleomorphic adenomas (PAs) and Warthin tumors (WTs) being the most common types, malignant transformation can occur, even in initially benign tumors like PAs [2]. This potential for malignancy makes surgical removal the preferred treatment [3].

Ultrasound (US) and magnetic resonance imaging (MRI) are the preferred modalities for evaluating PGTs due to their high diagnostic accuracy, lack of ionizing radiation, and ability to guide biopsy, with US being ideal for superficial lesions and MRI for deep or large tumors; in contrast, computed tomography (CT) and PET‐CT are reserved for specific indications such as staging, suspected malignancy with intracranial extension, or when MRI is contraindicated, due to their radiation exposure and lower soft tissue contrast [4].

In particular, US is the first‐line imaging technique—not only because of the gland's superficial anatomical location, but also due to its wide availability and ease of use [5]. The reported accuracy of B‐mode ultrasound in distinguishing between malignant and benign parotid gland tumors (PGTs) ranges from 57% to 96%, with sensitivity between 62% and 84%, and specificity between 88% and 96% [6, 7, 8]. The parotid glands' complex vascular network and the high vascularization of malignant tumors further complicate differentiation [9]. To enhance diagnostic precision, some authors advocate the use of ultrasound‐guided biopsy (USGB) to distinguish between benign and malignant lesions.

Nevertheless, logistical challenges often limit the use of these invasive techniques in clinical practice, and selecting patients in need of a USGB is not always easy [10]. Although it is a well‐established tool for preoperative diagnosis of salivary gland tumors, it can be technically challenging in the parotid gland. Deep lobe tumors, proximity to the facial nerve, and limited sonographic access can reduce feasibility, and relative contraindications such as anticoagulation must be considered. Core needle biopsy, in particular, requires more operator experience compared to fine needle aspiration to ensure safe and representative tissue sampling [11]. Accurate imaging‐based triage is therefore critical. According to the American Society of Clinical Oncology (ASCO) as well as the European Society for Medical Oncology (ESMO) Clinical Practice Guideline and the German AWMF‐S3‐Guideline for salivary gland malignancies, USGB (either fine needle aspiration cytology or core needle biopsy) is recommended when there is clinical or radiologic suspicion of malignancy, with the choice depending on lesion accessibility, risk profile, and institutional experience [12, 13, 14].

Another key imaging modality for the preoperative evaluation of parotid lesions is magnetic resonance imaging (MRI), as it helps determine whether a lesion is located in the superficial or deep lobe, assess tumor extent and signal characteristics, and evaluate the relationship to the facial nerve. While MRI has proven value in identifying malignant features such as irregular margins, perineural spread, and lymphadenopathy with a demonstration of its diagnostic value in a retrospective study of 133 patients, which reported a sensitivity of 90.3%, specificity of 77.5%, overall accuracy of 80.5%, positive predictive value of 54.9%, and negative predictive value of 96.3% [15], it is more time‐consuming and costly than ultrasound [16]. Therefore, some groups recommend MRI primarily when grayscale ultrasound provides insufficient information—such as in the case of very large tumors or those located in the deep parotid lobe [17].

The surgical approach in patients with a PGT depends on the tumor's nature, with varying levels of radicality for benign versus malignant cases [18, 19, 20, 21]. In benign PGTs, preserving the facial nerve is critical to maintain its function [22]. For malignant tumors, preoperative planning is essential to decide about the management of nerve infiltration and whether neck dissection is required. Patients must be informed about the potential consequences of nerve resection, such as facial weakness or paralysis, impaired facial expressions, difficulties with eye closure, speech and eating challenges, and synkinesis, including their psychosocial impact [23, 24]. Patients should be informed that reconstructive options are available, which ideally should happen during preoperative consultation [24, 25]. This proactive planning reduces the risk of complications [26], as repeat surgeries can be challenging due to extensive scarring [27].

Given the limitations of conventional US, this prospective study investigates the potential of the qualitative assessment of PGTs with multiparametric US (mpUS) to improve preoperative diagnostic accuracy for PGTs. MpUS integrates advanced techniques such as shear wave elastography (SWE) to assess tumor stiffness [28] and contrast‐enhanced US (CEUS) to evaluate microvascularization [29], alongside traditional B‐mode and color‐coded duplex sonography (CCDS).

While multiparametric imaging techniques such as MRI have been explored for PGTs, these approaches often rely on radiological infrastructure and postprocessing software [30]. In contrast, US remains the first‐line imaging modality in head and neck practice due to its accessibility, cost‐effectiveness, and ability to be performed directly by the surgeon [31].

Head and Neck surgeons frequently make intraoperative and outpatient decisions based on imaging they interpret themselves [32]. A qualitative mpUS approach allows immediate, clinically relevant interpretation without requiring quantitative analysis or external review, streamlining the diagnostic workflow.

Preliminary findings indicate that SWE and CEUS provide valuable insights: benign PGTs tend to show lower stiffness, while WTs exhibit higher perfusion compared to PAs, and malignant PGTs show distinct vascular patterns [33, 34, 35]. Our previous research focused on the quantitative evaluation of mpUS and demonstrated that CEUS intensity parameters—such as WiAUC, WoAUC, and WiWoAUC—can significantly distinguish between benign and malignant PGTs, particularly in differentiating Warthin's tumors from pleomorphic adenomas and squamous cell carcinomas [33].

However, the existing literature presents inconsistent findings regarding the diagnostic value of CEUS [35, 36]. While some studies support its discriminative potential based on perfusion parameters like AUC and MTT [37], others report no significant differences [38, 39], casting doubt on CEUS's standalone utility. Our preliminary results showed that, although CEUS revealed clear vascular distinctions between tumor types, SWE alone did not allow for reliable differentiation, reflecting both the promise and the limitations reported in earlier studies. These discrepancies are often attributed to methodological variability, particularly in ROI placement, imaging protocols, and tumor heterogeneity.

In light of these findings, we hypothesize that diagnostic accuracy can be further improved by applying qualitative criteria to mpUS interpretation, focusing on the interpretation of the vascularization patterns. The aim of this study is to evaluate the diagnostic accuracy of qualitative mpUS in distinguishing between benign and malignant parotid gland tumors (PGTs) and to develop a diagnostic workflow based on real‐time image interpretation—eliminating the need for post‐processing CEUS perfusion analysis—thereby enabling more accurate preoperative decision‐making and optimized selection of candidates for ultrasound‐guided biopsy (USGB).

## 2 Materials and Methods

### 2.1 Aim, Design and Setting of the Study

Before beginning the prospective inclusion of patients in this cohort study, we planned both a quantitative evaluation of mpUS datasets—to determine whether the new sonographic imaging method could identify differences between benign and malignant PGTs [33]—and a qualitative analysis to calculate diagnostic accuracies. At the time of study planning, our estimated sample size was based on two previously published diagnostic imaging studies: one evaluating 39 patients with PGTs using CEUS [9] and another assessing 57 tumors with elastography [40]. These studies demonstrated the feasibility and clinical relevance of CEUS and elastographic techniques in differentiating benign from malignant lesions. Following the end of patient enrollment and during manuscript preparation for the quantitative analysis, we retrospectively validated our sample size by referencing two more recent studies with larger cohorts: a CEUS study with 100 PGTs [41] and an elastography study including 104 tumors [42]. The similarity of our cohort size to these later publications supports the adequacy of our chosen sample size for evaluating the diagnostic performance of mpUS.

### 2.2 Study Population

Between August 2019 and September 2021, a consecutive series of patients was recruited by the outpatient clinic of the Otorhinolaryngology Department at Charité—Universitätsmedizin Berlin (Campus Charité Mitte) meeting the following inclusion criteria: (i) being older than 18 years, (ii) having a PGT of any size, and (iii) consenting to histological confirmation through surgery (partial or total parotidectomy) or US‐guided USGB. Tumors located in the deep lobe of the parotid gland were excluded from the study due to known limitations of ultrasound imaging in this region, where acoustic shadowing and reduced penetration may impair image quality and diagnostic accuracy [43]. Ninety‐six patients met these criteria and 98 PGTs were included in this study, as two patients underwent surgery on both sides. One patient was not able to have surgery due to anesthesiologic reasons, and histological confirmation was gained through USGB. The study was approved by the institutional ethics committee of Charité‐Universitätsmedizin Berlin (protocol code EA1/087/19, date of approval: 2 May 2019), and written informed consent was obtained from all participants in accordance with the Declaration of Helsinki of 1975 as revised in 1983.

### 2.3 US Protocol and Interpretation

For the index test, all participants underwent an US‐examination of the neck using the same device, probe, and setup (4–10 MHz multifrequency linear array transducer with a center frequency of 7 MHz, Acuson Sequoia, Siemens Healthineers, Erlangen, Germany) prior to surgery or USGB. US was performed according to a standard protocol by two examiners (one ENT‐specialist (EFSUMB Head and Neck Level II) and one radiologist (EFSUMB Level II)) with extensive experience in neck US. The US protocol included B‐mode US, color‐coded duplex sonography (CCDS), shear wave elastography (SWE), and contrast‐enhanced US (CEUS).

During SWE, five consecutive images were obtained, and for the purpose of the quantitative assessment presented in a previous study [33], a circular region of interest (ROI) adapted to the size of the PGT was placed in dual‐image mode—displaying the B‐mode image on the left and the color‐coded SWE map on the right—with the ROI visualized in both images for full lesion assessment. Propagation velocity within the ROI was measured in meters per second, and a color‐coded elastogram was generated, which displays tissue stiffness in a continuum of colors [44]. The acquisition of five consecutive measurements was performed to calculate the mean SWE velocity, allowing for quantification and minimization of inter‐frame variability, and thereby enhancing the robustness and reproducibility of the quantitative data. However, one limitation of quantitative SWE is that values cannot be reliably compared across different device manufacturers, rendering cut‐off values inconsistent between systems [42]. For this reason, we opted to use the qualitative assessment of the color‐coded elastogram rather than relying on quantitative cut‐off value [42]. Our device uses the following colors to encode different levels of tissue stiffness:

- *Blue*: Soft—Areas with low resistance, indicating high elasticity.
- *Green*: Moderately soft—Areas with slightly increased resistance, showing reduced elasticity.
- *Yellow*: Hard—Areas with significantly reduced elasticity, indicating greater stiffness.
- *Red*: Very hard—Areas with minimal elasticity or maximum resistance, indicating extreme stiffness.

For CEUS examination, patients were positioned supine with the head slightly extended and turned contralaterally to the side of the lesion to optimize exposure of the parotid region. A low mechanical index (< 0.1) setting was used to preserve microbubble integrity during the examination. A second‐generation US contrast agent (SonoVue, Bracco Imaging, Milan, Italy) was administered as a bolus injection of 2.4 mL via a peripheral vein, immediately followed by a 10 mL flush of 0.9% sodium chloride, injected at a steady rate of approximately 1 mL/s. From the onset of injection, a 90‐s cine loop was recorded to capture dynamic enhancement, including arterial inflow and washout phases, which were stored in DICOM format for consistent playback and evaluation. The region of interest was continuously monitored in real time, and care was taken to avoid excessive probe pressure during the examination. The contrast agent contains microbubbles filled with sulfur hexafluoride and is eliminated through the lungs within 10 to 15 min. It has a very low incidence of adverse events (0.088%), with headache or nausea being the most common mild side effects (0.047%) [45].

Specific CEUS parameters such as wash‐in rate, wash‐out rate, and time to peak were not evaluated in this study, as the focus was on qualitative assessment; however, these quantitative perfusion metrics were analyzed in detail in our previous study on the quantitative evaluation of mpUS [33].

Artifacts were minimized by adhering to standardized acquisition protocols. Specifically, excessive probe pressure was avoided, patients were asked to remain still, and a low mechanical index was maintained throughout the CEUS recording to preserve microbubble stability. In cases where motion or contrast‐related artifacts occurred, cine loops were either repeated or excluded from further evaluation.

An irregular border in B‐mode US was interpreted to indicate malignancy. CCDS findings were qualitatively assessed based on vascularization patterns, following the classification proposed by Mansour et al. [39] (2017). Vascularization was categorized as weak or absent or strong, with both internal and peripheral components in malignant, and moderate and peripheral vascularization in benign PGTs. Although CCDS patterns alone are not universally established as reliable malignancy criteria in PGTs, we aimed to evaluate their diagnostic utility independently in this study. Therefore, CCDS was analyzed as an isolated parameter, using the qualitative pattern definitions described above.

In the qualitative assessment of SWE, tumors were evaluated based on their relative stiffness compared to the surrounding parotid tissue using the color‐coded elastogram. Lesions that appeared softer or showed homogeneous elasticity similar to the adjacent glandular parenchyma were classified as likely benign. Lesions that appeared clearly harder than the surrounding tissue were considered potentially suspicious, but not automatically malignant. This classification was made in reference to prior literature noting that certain benign tumors—particularly pleomorphic adenomas and Warthin tumors—may also demonstrate increased stiffness [42, 46]. Therefore, increased stiffness alone was not used as a definitive malignancy criterion, but rather as a supportive feature to be interpreted in combination with B‐mode and CEUS findings. This approach reflects the known overlap in elastographic characteristics between benign and malignant parotid gland tumors and underscores the multimodal nature of the mpUS assessment. Note that the velocity measured in the ROI of each SWE image was not used here but in an earlier study of quantitative measures [33].

Microvascular density is another crucial feature for lesion characterization and can be assessed using CEUS. Neoangiogenesis, the process by which a benign tumor increases its blood supply, is an indicator of potential malignant transformation and might develop into abnormal angiogenesis, which disregards normal patterns of vessel formation [47, 48] and may initially result in densely packed microvessels leading to hyperenhancement in CEUS, but eventually causes microvascular deficiencies over time [47].

Absent enhancement in CEUS was interpreted in conjunction with the B‐mode‐US findings. The absence of enhancement in CEUS can indicate cystic components in benign lesions [49, 50] but can also suggest necrotic areas in malignant PGTs [51, 52, 53]. We thus interpreted regions lacking microvascularization in PGTs with regular borders as benign and in PGTs with irregular borders as malignant [33], in line with malignancy criteria described by other study groups [39, 53, 54]. The rating criteria are summarized in Table 1.

**TABLE 1:** Rating criteria used for interpretation of mpUS datasets.

US mode	Benign	Malignant
B‐mode US	Regular borders	Irregular borders
CCDS	Moderate peripheral vascularization	Weak or absent vascularization Strong internal and peripheral vascularization
SWE	Elastogram: same color of PGT and surrounding parotid gland OR PGT softer than surrounding tissue (blue = soft, green = moderately soft, yellow = hard, red = very hard)	Elastogram: PGT harder than parotid gland tissue (blue = soft, green = moderately soft, yellow = hard, red = very hard)
CEUS		
Enhancement	Hypoenhancement	Hyperenhancement
Regions lacking microvascularization	In conjunction with regular borders in B‐mode US	In conjunction with irregular borders in B‐mode US

Abbreviations: CCDS = color‐coded duplex sonography; CEUS = contrast‐enhanced ultrasound; PGT = parotid gland tumor; SWE = shear wave elastography; US = ultrasound.

Histopathological results were used as the reference standard to validate the findings obtained with the mpUS examination used here.

### 2.4 Image Interpretation and Diagnostic Workflow

Images from all four ultrasound modalities (B‐mode, CCDS, SWE, CEUS) were reviewed in consensus by an experienced ENT specialist (EFSUMB Level II, head and neck) and a radiologist (EFSUMB Level III). In case of differing impressions, a final consensus was reached through immediate discussion. Interpretations were based on predefined qualitative criteria from the literature and clinical experience (see Table 1), reflecting real‐world diagnostic practice. Image interpretation criteria for SWE and CEUS were based on previously published standards; no separate pilot testing for inter‐reviewer consistency was performed in this study. While no formal scoring system was used, we acknowledge the need for standardized criteria in future research to improve reproducibility.

Each modality was first assessed individually, and diagnostic performance metrics (sensitivity, specificity, PPV, NPV, and accuracy) were calculated separately. In a second step, all image sets were re‐evaluated in consensus to generate an overall mpUS classification. This integrated rating reflected the relative diagnostic contribution of each modality, based on both our results and prior literature, and was intended to simulate a real‐world diagnostic workflow.

The two reviewers were not involved in image acquisition and were blinded to histopathological results. Both were highly experienced in head and neck US. Because all assessments were performed in consensus, no inter‐reader reliability analysis was conducted. The pathologists defining the reference standard had access to clinical information.

### 2.5 Statistical Analysis

The mpUS dataset evaluated in consensus by two readers and the histopathological reference standard of each patient was analyzed to assess diagnostic performance, evaluating sensitivity, specificity, PPV, NPV, and accuracy using cross tables. The diagnostic assessment focused on distinguishing between benign and malignant parotid gland tumors, as this distinction directly impacts surgical planning in cases with suspected malignancy. A two‐sided significance level of *α* = 0.05 was set to indicate statistical significance. Relationships between categorical variables were tested using Pearson's chi‐square test. Receiver operating characteristic (ROC) curves were generated for each US modality (B‐mode, CCDS, SWE, and CEUS) to visualize diagnostic performance, and the area under the curve (AUC) was calculated to assess overall discriminative ability. All statistical analyses were conducted using SPSS software (IBM Corp. Released 2016. IBM SPSS Statistics for Windows, Version 27.0. Armonk, NY, USA: IBM Corp.). No missing datasets for the reference standard results were reported, and no explorative analyses were conducted. In the case of missing datasets, participants were excluded from analysis.

## 3 Results

### 3.1 Study Population

**FIGURE 1:**
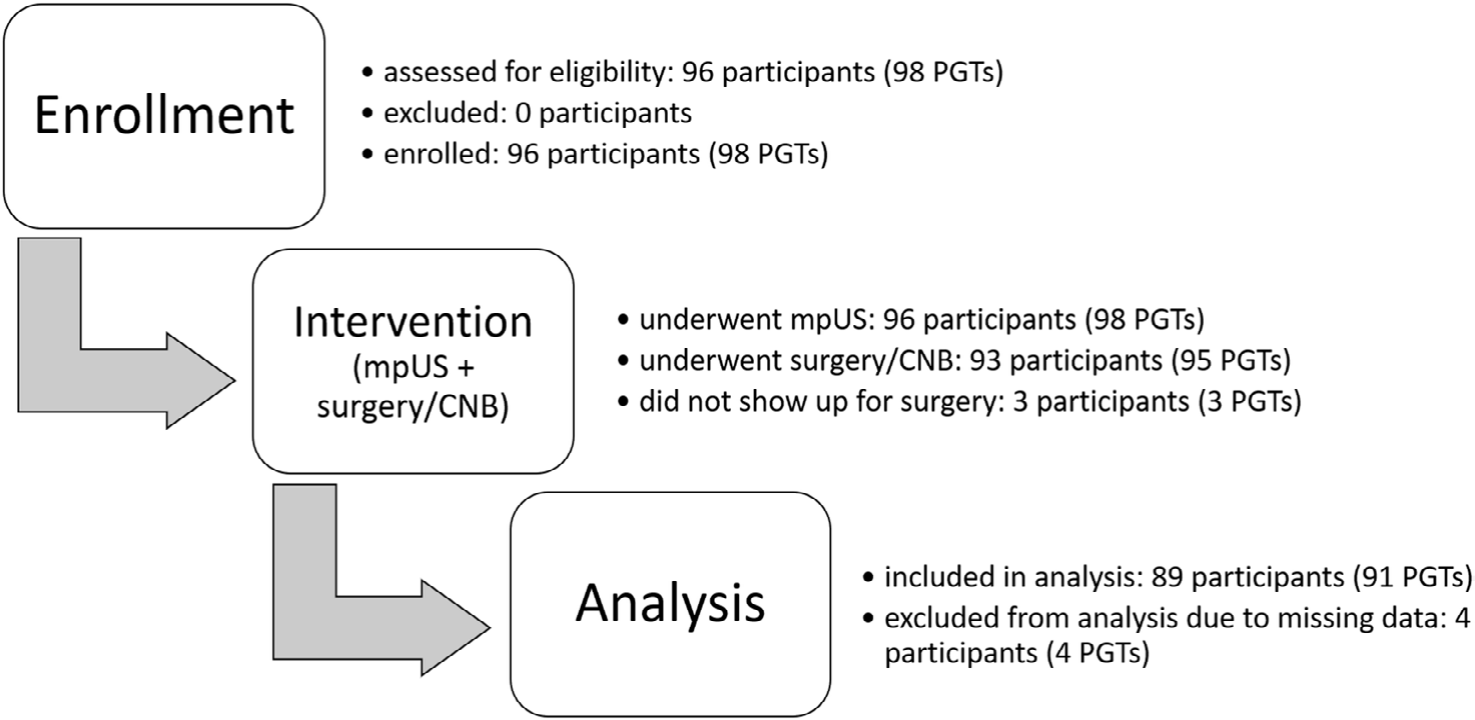
Flow of participants. mpUS = multiparametric ultrasound; PGTs = parotid gland tumors; USGB = ultrasound guided biopsy.

Ninety‐six participants with 98 PGTs were initially enrolled. Three participants (with three PGTs) did not show up for surgery and were therefore excluded from analysis. For technical reasons, image datasets of four participants (with four PGTs) could not be stored and thus were not included in the analysis, leaving us with 89 participants and 91 PGTs for analysis. Of the 89 participants, 32 were female (35.96%) and of the 91 PGTs, 24 were malignant (26.37%). The flow of participant inclusion and exclusion is presented in Figure 1 and the baseline demographic data are presented in Table 2.

**TABLE 2:** Baseline characteristics of the study population, including age, sex distribution, tumor dimensions in centimeters (length, width, height), and the distribution of different benign and malignant parotid gland tumors.

Characteristic	
Age (years)	62.1 (+/− 18.0)
Sex (male/female)	66/32
Tumor length	1.9 (+/− 0.77) cm
Tumor width	1.42 (+/− 0.67) cm
Tumor height	1.96 (+/− 0.83) cm

Histopathological result	
Benign	67
Pleomorphic adenoma	35
Warthin tumors	20
Lymph nodes	7
Other benign lesions (cysts, oncocytoma)	5
Malignant	24
Squamous cell carcinoma	12
Adenocarcinoma	4
Lymphoma	4
Malignant melanoma	3
Mucoepidermoid carcinoma	1
Adenoid cystic carcinoma	1
Merkel cell carcinoma	1

*Note:* Values are reported as mean ± standard deviation or absolute counts.

### 3.2 Test Results

**FIGURE 2:**
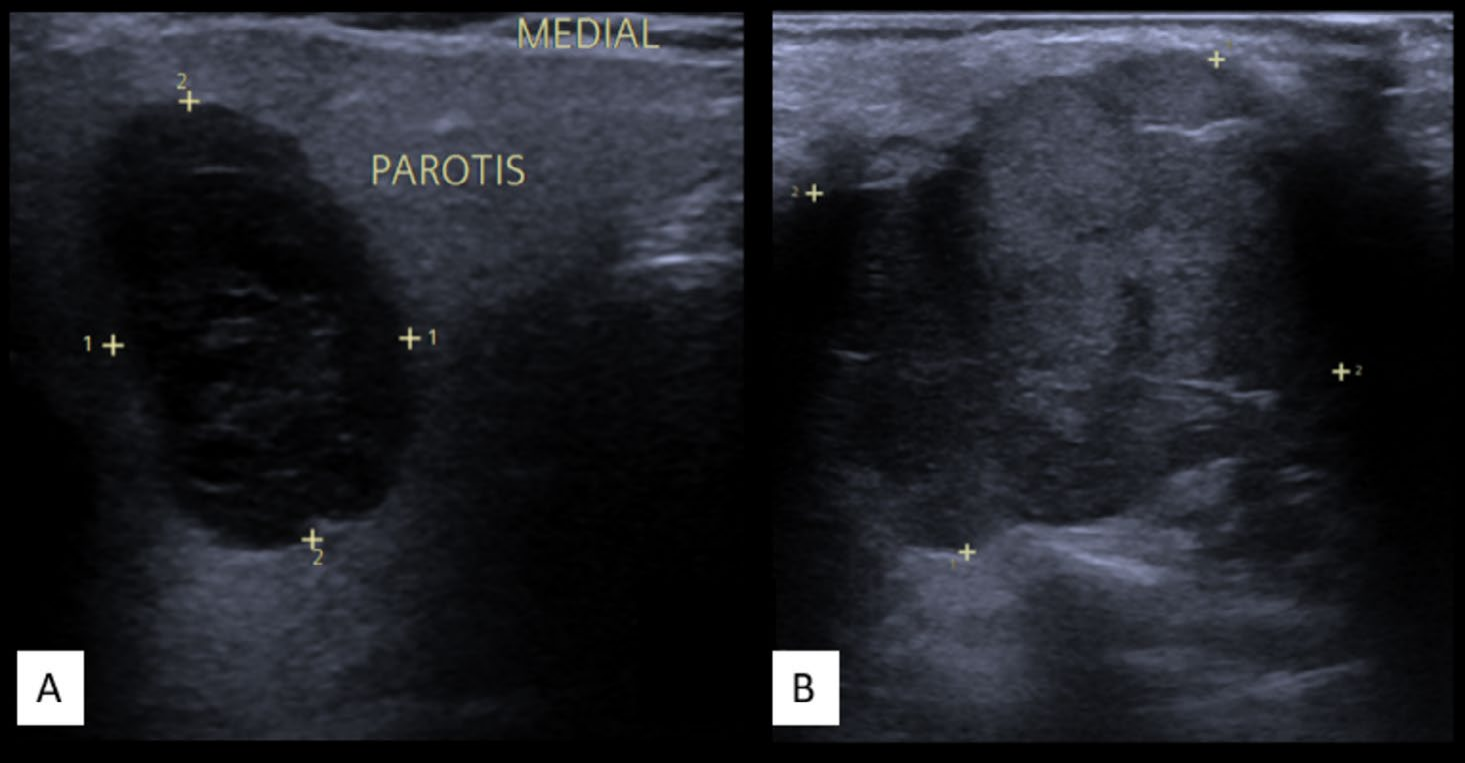
Example of B‐Mode‐US. (A) Benign parotid gland tumor with regular borders. (B) Malignant parotid gland tumor with irregular borders. Please note that since only qualitative parameters were evaluated, tumor size was excluded. This also applied to the two‐blinded experts who assessed the lesions. [Color figure can be viewed at wileyonlinelibrary.com]

B‐mode US demonstrated good diagnostic performance with a sensitivity of 71% (49.1–86.9), specificity of 87% (75.0–94.1), PPV of 65% (44.3–81.9), NPV of 89% (77.4–95.4), and accuracy of 82% (72.3–89.2) (*χ* ^2^ (1) = 28.528, *p* < 0.001). Refer to Figure 2 for an example.

SWE displayed moderate performance with a sensitivity of 67% (45.2–83.9), specificity of 70% (56.3–81.5), PPV of 44% (27.2–62.1), NPV of 85% (71.1–93.3), and accuracy of 69% (58.7–78.1) (*χ* ^2^ (1) = 17.025, *p* = 0.002).

**FIGURE 3:**
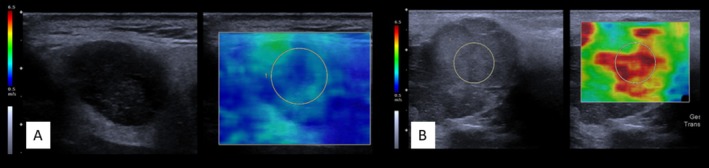
Example of shear wave elastography. On the left side of each example is the B‐mode US image of the lesion; on the right is the color‐coded representation based on shear wave velocity. See the elastogram on the left side of each image: Blue indicates low, and red indicates high shear wave velocity, serving as an indicator of tissue stiffness. Please note that the actual shear wave velocity values (measured in m/s) were excluded, as the focus was solely on evaluating qualitative imaging features. The two blinded experts assessing the images did not have access to the velocity values. (A) Benign parotid gland tumor, with the tumor and the gland parenchyma showing nearly the same colors. (B) Malignant parotid gland tumor, with a clear color difference between tumor and gland. [Color figure can be viewed at wileyonlinelibrary.com]

An example is shown in Figure 3.

**FIGURE 4:**
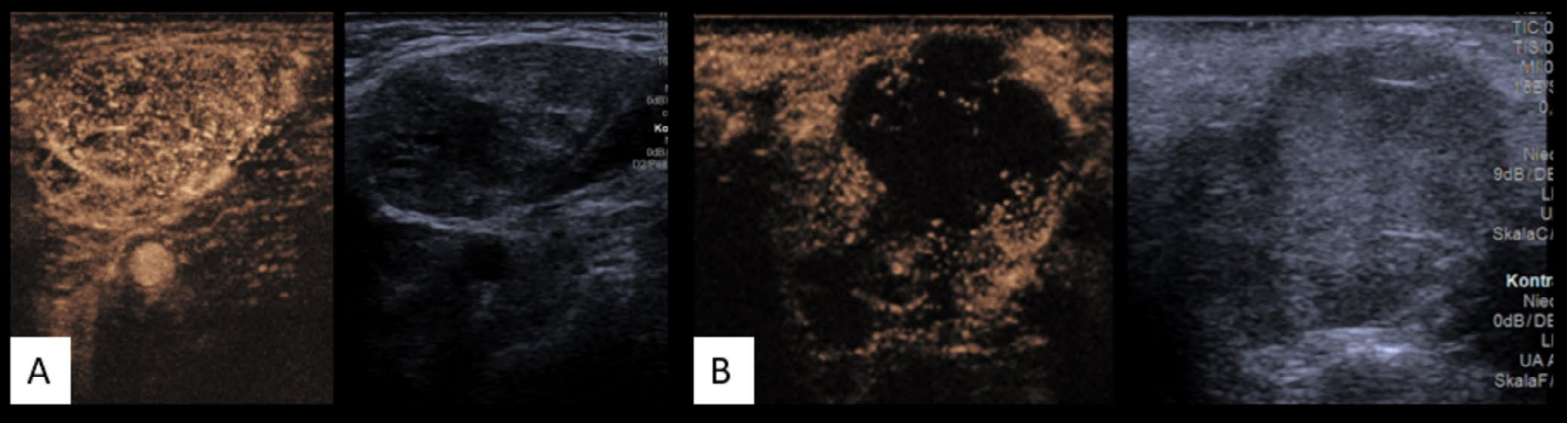
Examples of CEUS using still images. On the left, a frame from the CEUS loop; on the right, the corresponding B‐mode image to confirm localization during the loop. (A) Benign parotid tumor with strong, regular contrast enhancement. (B) Malignant tumor with intense peripheral enhancement and larger central contrast defects, indicating avascular areas. [Color figure can be viewed at wileyonlinelibrary.com]

CEUS exhibited high sensitivity (83%, 95% CI: 62.6–94.0) but lower specificity (66%, 51.7–77.9), with a PPV of 47% (31.2–63.5), NPV of 92% (79.6–97.6), and accuracy of 70% (59.0–79.3) (χ^2^(1) = 10.017, *p* < 0.001). See Figure 4 for an example.

**FIGURE 5:**
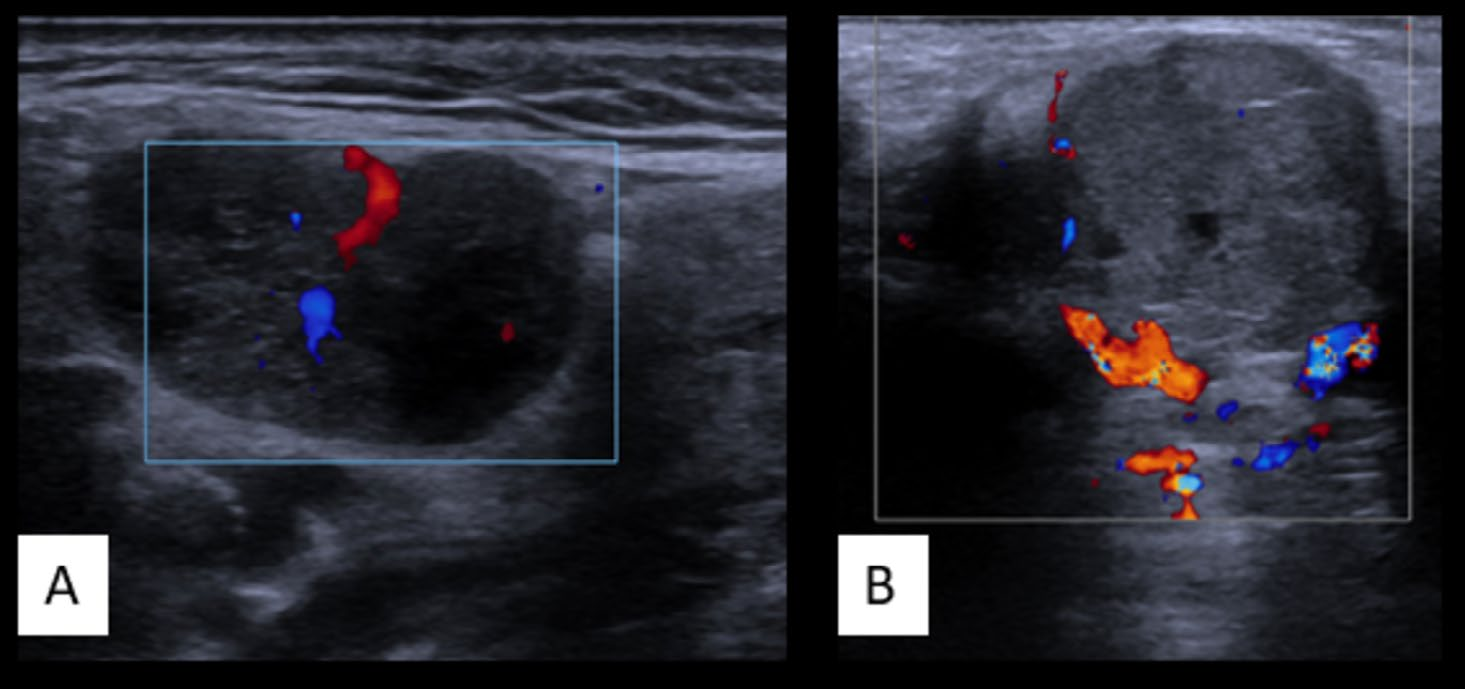
Examples of CCDS using still images. (A) Benign parotid gland tumor with central vascularization. (B) Malignant lesion with peripheral (rim) vascularization. [Color figure can be viewed at wileyonlinelibrary.com]

CCDS showed the lowest diagnostic performance with a sensitivity of 50% (30.7–69.3), specificity of 67% (53.4–78.3), PPV of 35% (20.6–52.9), NPV of 79% (65.5–88.3), and accuracy of 62% (51.0–72.0), which was not statistically significant (*χ* ^2^ (1) = 22.224, *p* > 0.05). Examples are shown in Figure 5.

Our results indicate that mpUS has high diagnostic performance for characterizing PGTs. We found a sensitivity of 83% (95% CI: 62.6–94.0), specificity of 94% (84.3–98.1), PPV of 83% (62.6–94.0), NPV of 94% (84.3–98.1), and overall accuracy of 91% (82.4–96.3) (*χ* ^2^ (1) = 54.464, *p* < 0.001).

Results are compiled in Table 3.

**TABLE 3:** Diagnostic performance results for mpUS and the individual ultrasound techniques are presented, with sensitivity, specificity, PPV, NPV, and accuracy given as percentages, along with 95% confidence intervals, corresponding *p* values, and chi‐squared values (degrees of freedom indicated in brackets).

US mode	Sensitivity	Specificity	PPV	NPV	Accuracy	*χ* ^2^	*p*
B‐mode US	71%	87%	65%	89%	82%	*χ* ^2^ (1) = 28.528	< 0.001
	(0.51–0.85)	(0.76–0.93)	(0.46–0.81)	(0.79–0.95)	(0.73–0.89)		
CCDS	67%	70%	44%	85%	69%	*χ* ^2^ (1) = 22.224	> 0.05
	(0.47–0.82)	(0.71–0.89)	(0.39–0.73)	(0.77–0.93)	(0.68–0.85)		
SWE	83%	66%	47%	92%	70%	*χ* ^2^ (1) = 17.025	0.002
	(0.64–0.93)	(0.54–0.76)	(0.33–0.61)	(0.80–0.97)	(0.60–0.79)		
CEUS	50%	67%	35%	79%	62%	*χ* ^2^ (1) = 10.017	< 0.001
	(0.31–0.69)	(0.55–0.77)	(0.21–0.52)	(0.67–0.88)	(0.52–0.72)		
mpUS	83%	94%	83%	94%	91%	*χ* ^2^ (1) = 54.464	< 0.001
	(0.64–0.93)	(0.86–0.98)	(0.64–0.93)	(0.86–0.98)	(0.84–0.95)		

Abbreviations: *χ* ^2^ = Pearson's chi‐square test; CCDS = color‐coded duplex sonography; CEUS = contrast‐enhanced ultrasound; mpUS = multiparametric ultrasound; NPV = negative predictive value; PPV = positive predictive value; SWE = shear wave elastography; US = ultrasound.

### 3.3 ROC Curve Analysis

**FIGURE 6:**
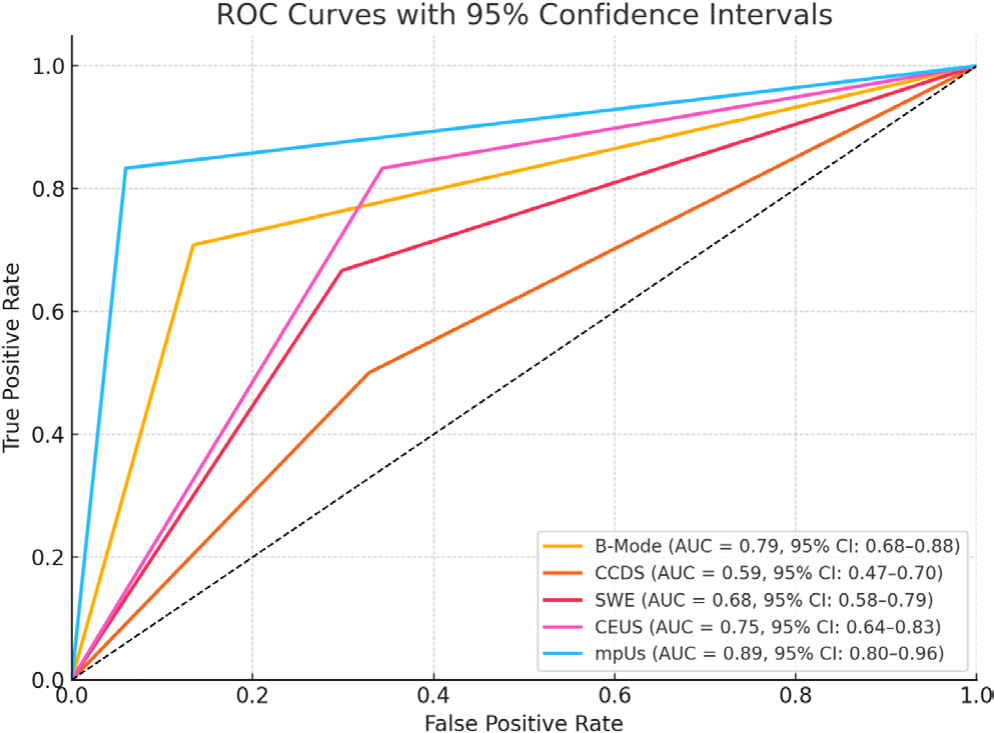
ROC curves comparing the diagnostic performance of B‐mode, CCDS, SWE, CEUS, and mpUS in distinguishing benign from malignant parotid gland tumors. AUC = area under the curve; 95% confidence intervals are provided in parentheses. [Color figure can be viewed at wileyonlinelibrary.com]

ROC curves were generated for B‐mode, CCDS, SWE, CEUS, and mpUS using histopathology as the reference standard. The analysis demonstrated that mpUS achieved the highest diagnostic performance, with an AUC of 0.887 (95% CI: 0.802–0.959). B‐mode showed an AUC of 0.787 (95% CI: 0.683–0.880), followed by CEUS with an AUC of 0.745 (95% CI: 0.642–0.831) and SWE with an AUC of 0.684 (95% CI: 0.578–0.787). CCDS had the lowest discriminative ability, with an AUC of 0.586 (95% CI: 0.467–0.696). These findings further support the superior diagnostic accuracy of mpUS compared to individual ultrasound techniques. The ROC curves are shown in Figure 6. These findings support the diagnostic performance results reported earlier and visually illustrate the trade‐offs between sensitivity and specificity for each modality.

## 4 Discussion

The results of this study provide a detailed evaluation of various US modalities used for diagnosing PGTs and underscore the superior performance of mpUS compared with individual techniques alone.

B‐mode‐US alone also showed good diagnostic performance with 82% accuracy, 71% sensitivity, and 87% specificity. Its high NPV of 89% suggests that it can effectively rule out malignancy. However, its lower PPV (65%) indicates limitations in definitively confirming malignancy. While valuable as a baseline imaging modality, B‐mode‐US alone may not be sufficient for precise differentiation of PGTs and benefits significantly from the additional use of advanced US techniques.

SWE demonstrated moderate diagnostic performance, with 69% accuracy, 67% sensitivity, and 70% specificity. Its NPV of 85% indicates reasonable reliability in ruling out malignancy, but its lower specificity and PPV (44%) limit its ability to confirm malignancy. SWE is most effective as a supplementary tool when combined with other methods, particularly in cases where stiffness characteristics provide additional diagnostic insights.

In the present study, CEUS was assessed using a qualitative approach based on real‐time visual interpretation of enhancement patterns, which mirrors routine clinical workflow and allows immediate diagnostic decision‐making.

CEUS showed high sensitivity (83%) and an excellent NPV (92%), making it effective in both confirming and ruling out malignancy. However, its lower specificity (66%) and PPV (47%) indicate a tendency toward overestimation of malignancy, leading to false positives. CEUS is therefore particularly valuable for identifying malignancy but should be interpreted alongside other modalities to reduce overdiagnosis. This is in line with previous studies.

CCDS emerged to have the lowest diagnostic accuracy (62%), with both poor sensitivity (50%), and specificity (67%) in our study. It failed to achieve statistical significance, making it the least reliable method for characterizing PGTs in our cohort. CCDS has limited standalone diagnostic value and is best used as a supplementary tool alongside other techniques.

This trend was further supported by ROC curve analysis, where CEUS and B‐mode yielded the highest AUCs, reinforcing their potential as front‐line imaging tools in PGT characterization.

Among all techniques evaluated, the combined approach referred to as “mpUS” demonstrated the highest diagnostic accuracy at 91%, with a sensitivity of 83% and a specificity of 94%. The high PPV and NPV suggest that mpUS may offer reliable support in distinguishing malignant from benign parotid tumors. These findings indicate that mpUS could be a valuable tool for preoperative evaluation, potentially reducing diagnostic uncertainty and contributing to more informed surgical planning.

The high diagnostic accuracy of mpUS can significantly enhance preoperative planning and patient management by improving differentiation of benign and malignant PGTs. MpUS could thus help in noninvasively determining the optimal management of PGTs in patients with significant perioperative risks such as morbid obesity or advanced age [55]. A diagnostic modality that improves the differentiation of PGTs may help reduce unnecessary surgical procedures for benign tumors, sparing these high‐risk patients invasive interventions and their associated complications such as cognitive impairment or respiratory failure [56, 57, 58]. This is particularly valuable for patients with significantly elevated perioperative risks, where careful decision‐making is essential to balance diagnostic and therapeutic goals with patient safety [59]. Additionally, mpUS's ability to identify malignant tumors facilitates early and accurate planning for radical surgical approaches, including the management of potential facial nerve involvement and neck dissection. Nevertheless, such decisions should not be made based on imaging alone but need histological confirmation.

**FIGURE 7:**
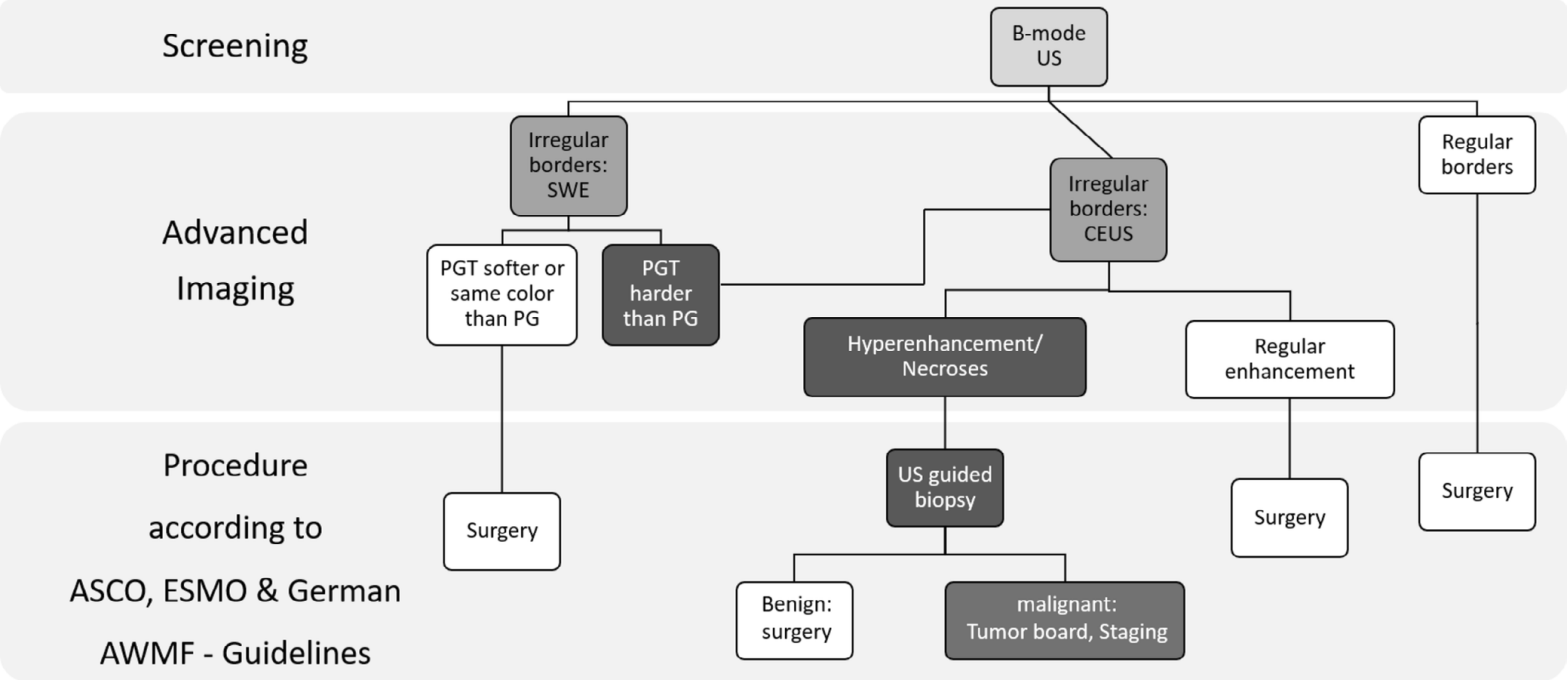
Proposed diagnostic pathway for the evaluation of parotid gland tumors using multiparametric ultrasound (mpUS). The pathway integrates B‐mode ultrasound, shear wave elastography (SWE), and contrast‐enhanced ultrasound (CEUS). SWE is used as a supportive tool to further assess lesions that are indeterminate on B‐mode. Lesions that appear clearly soft on elastography may proceed directly to surgery, whereas those with increased stiffness are evaluated further with CEUS. SWE does not act as a standalone criterion but supports clinical decision‐making within a stepwise, multimodal diagnostic strategy. The proposed imaging workflow is designed to support structured decision‐making and has been combined with current ASCO, ESMO, and German AWMF guideline recommendations, which advocate ultrasound‐guided biopsy in cases of suspected malignancy based on clinical or radiologic findings. CEUS = contrast‐enhanced ultrasound; PG = parotid gland; PGT = parotid gland tumor; SWE = shear wave elastography; US = ultrasound.

**FIGURE 8:**
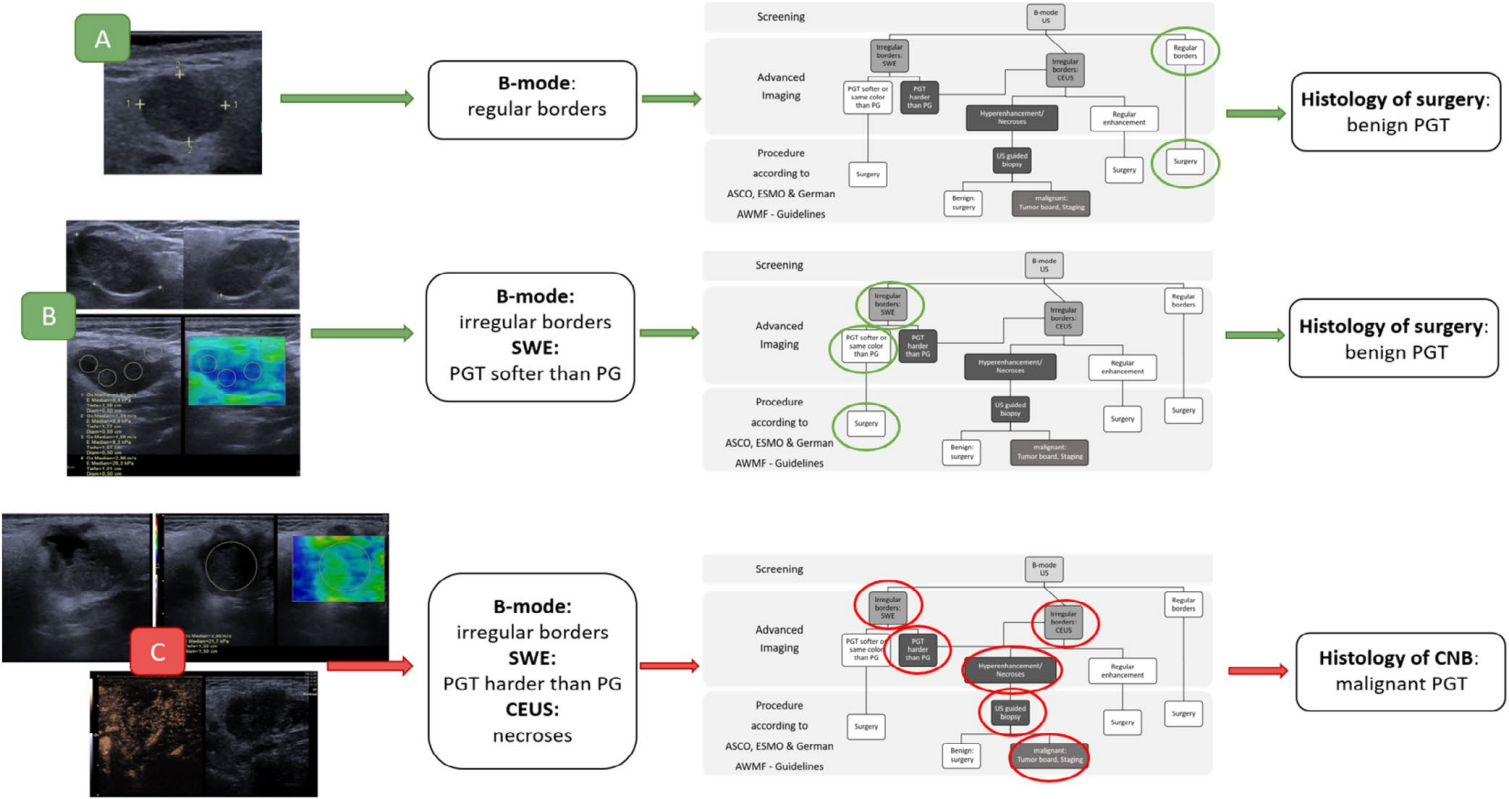
Examples showing how the imaging‐based workflow can support clinical decision‐making in accordance with ASCO, ESMO, and German AWMF guideline recommendations after imaging. Example A: A parotid gland tumor with regular borders on B‐mode ultrasound (US), suggesting a low likelihood of malignancy. Example B: A parotid gland tumor with irregular borders on B‐mode ultrasound (US) prompted a recommendation for advanced imaging. Despite its irregular appearance, the tumor exhibits a blue (soft) signal, while the surrounding gland tissue appears green (slightly harder), suggesting that malignancy is unlikely. Example C: A parotid gland tumor with irregular borders on B‐mode ultrasound prompts further evaluation with advanced imaging. The lesion demonstrates increased stiffness compared to the surrounding parotid tissue on shear wave elastography, warranting contrast‐enhanced ultrasound (CEUS). CEUS reveals hyperenhancement with areas lacking microvascularization, suggestive of necrosis. In accordance with the ASCO, ESMO, and German AWMF guidelines, an ultrasound‐guided biopsy (USGB) was performed, confirming malignancy. The case was subsequently staged and discussed in an interdisciplinary tumor board, leading to the decision to proceed with a more extensive surgical approach. CEUS = contrast‐enhanced ultrasound; PG = parotid gland; PGT = parotid gland tumor; SWE = shear wave elastography; US = ultrasound; USGB = ultrasound guided biopsy. [Color figure can be viewed at wileyonlinelibrary.com]

We therefore recommend a diagnostic pathway that we derived from our results (see Figures 7 and 8), incorporating B‐mode US, SWE, and CEUS—the modalities that demonstrated the highest diagnostic utility—while excluding CCDS due to its limited diagnostic performance and lack of added clinical value in our cohort.

In this pathway, lesions with benign‐appearing features (e.g., regular margins, soft SWE pattern, regular CEUS enhancement) are classified as having no suspicion of malignancy. In contrast, lesions showing irregular margins, increased stiffness, or atypical enhancement patterns are classified as suspicious for malignancy.

Importantly, the pathway does not prescribe clinical decisions such as whether to perform a biopsy or proceed with surgery. Instead, it ends at the level of imaging‐based suspicion. Any further diagnostic or therapeutic steps should be determined in accordance with established clinical guidelines and multidisciplinary evaluation. However, the implication of suspicious imaging is in line with the ASCO, ESMO, and German AWMF guidelines, which support ultrasound‐guided biopsy (USGB) in cases of suspected malignancy to obtain a preoperative histologic diagnosis [12, 13]. Accordingly, we have implemented this recommendation into the diagnostic pathway to ensure alignment with evidence‐based practice while maintaining the focus on imaging‐driven decision support.

Although SWE demonstrated only moderate diagnostic performance in this study, it was deliberately included in our diagnostic pathway due to its utility in clinical triage. SWE allows for rapid, non‐invasive assessment of tissue stiffness without the need for contrast administration. While not definitive on its own, qualitative elastogram interpretation—particularly when a lesion appears clearly soft—can support a benign impression and may help avoid unnecessary CEUS. Conversely, increased stiffness raises suspicion and justifies further evaluation with CEUS. Thus, SWE serves as a pragmatic intermediate step in the mpUS algorithm. This stepwise approach is intended to enhance diagnostic accuracy while maintaining efficiency and clinical applicability.

### 4.1 Initial Screening

B‐mode US:

Perform B‐mode US to evaluate parotid gland tumors (PGTs) for size, shape, borders, and internal echotexture.

Outcomes:

– If findings are clearly benign (e.g., smooth borders, homogeneous structure), proceed directly to surgical planning.
– If findings are unclear or raise suspicion for malignancy (e.g., irregular margins, heterogeneous texture), continue with advanced ultrasound techniques.

### 4.2 Advanced Imaging Techniques

SWE:

Assess the stiffness of the lesion in comparison to surrounding parotid tissue.

– Low stiffness: Suggests benignity; proceed with surgical planning as appropriate.
– High stiffness or heterogeneous elastogram: Raises concern for malignancy; proceed to CEUS evaluation.

CEUS:

Evaluate vascularization and perfusion characteristics.

– Regular, homogeneous enhancement: Likely benign; proceed with surgery.
– Irregular enhancement, hyperenhancement, or non‐enhancing (necrotic) areas: May indicate malignancy; consider biopsy.

### 4.3 Decision to Perform Ultrasound‐Guided Biopsy (USGB)

ASCO, ESMO, and German AWMF guidelines recommend performing USGB (FNA or CNB) when malignancy is suspected based on imaging or clinical findings.

Perform USGB if any of the following apply:

– Imaging shows high stiffness on SWE or irregular/atypical vascularization on CEUS.
– Advanced imaging is inconclusive or shows overlapping benign and malignant features.
– A precise preoperative diagnosis is needed due to clinical context (e.g., advanced age, comorbidities, or patient preference).
– There are additional clinical indicators of malignancy such as rapid tumor growth, pain, or facial nerve dysfunction.

### 4.4 Surgical Management Based on Biopsy Results (According to ASCO, ESMO, and German AWMF Guidelines)

Benign diagnosis on USGB:

– Proceed with planned conservative surgery while preserving facial nerve function.
– Further staging is not routinely necessary unless new findings arise.

Malignancy confirmed on USGB:

– Initiate cross‐sectional imaging (e.g., MRI, CT) and perform full staging workup.
– Plan radical parotidectomy with appropriate nerve management.
– Neck dissection should be considered based on tumor type, grade, and guideline recommendations.
– All malignant cases should be discussed in a multidisciplinary tumor board before surgery.

If malignancy is found only postoperatively:

– Stage the patient after surgery.
– Consider delayed neck dissection or re‐excision if margins are inadequate, based on tumor board recommendation.

This staged, imaging‐driven approach ensures diagnostic clarity and aligns with ASCO, ESMO, and German AWMF guidelines on imaging‐guided preoperative planning for malignant salivary gland tumors [12].

Our diagnostic pathway for PGTs builds upon prior multimodal ultrasonographic approaches and introduces a refined, streamlined decision‐making algorithm tailored for real‐world clinical use. When considered alongside the ASCO, ESMO, and German AWMF guideline recommendations [12, 13, 14], the pathway offers structured imaging‐based support that may help inform decisions regarding further diagnostic steps such as USGB, which shows similarities to a proposed comprehensive diagnostic model incorporating clinical examination, B‐mode US, CCDS, real‐time elastography, and CEUS, supported by perfusion parameters such as mean transit time (nMTT) and elastographic stiffness grading by Mansour et al. (2017). In their approach, malignancy suspicion was based on the presence of at least two of several predefined imaging and clinical features.

In contrast, our pathway focuses solely on assessing whether a parotid gland tumor appears suspicious for malignancy based on imaging findings, and can thereby support planning of further management steps as recommended by the ASCO, ESMO, and German AWMF guidelines. While both pathways integrate B‐mode US, SWE, and CEUS, we excluded CCDS from our final decision‐making algorithm due to its low sensitivity and lack of statistical significance in our data.

While SWE showed only moderate diagnostic performance on its own, it was retained in the proposed diagnostic pathway due to its potential added value when interpreted alongside B‐mode and CEUS. SWE provides additional information on tissue stiffness, which may help support imaging‐based suspicion in certain cases, particularly when other modalities yield inconclusive findings. Its non‐invasive nature and ease of integration into routine ultrasound exams make it a practical component of a multiparametric approach. Although its individual contribution is limited, SWE may contribute to a more comprehensive overall assessment when used as part of a stepwise imaging strategy.

Our protocol relies on qualitative interpretation of SWE and CEUS by two experienced readers in real time, aiming to reflect everyday clinical settings without the need for postprocessing tools.

One of the strengths of the study by Mansour et al. is its large sample size of 202 parotid gland lesions, which adds weight to the evidence supporting mpUS as a valuable diagnostic tool. Their findings further reinforce our results, suggesting that mpUS is well suited for inclusion in structured diagnostic pathways.

While Mansour's model emphasizes structured scoring and quantitative perfusion analysis (e.g., using the postprocessing software VueBox), our approach emphasizes simplicity, accessibility, and diagnostic efficiency—making it feasible for use in clinical environments where such software may not be available. Ultimately, both models highlight the diagnostic potential of mpUS, and future studies should directly compare their clinical impact, cost‐effectiveness, and reproducibility across multiple centers.

These findings seem to highlight the superiority of mpUS as the most accurate and reliable diagnostic modality, integrating the strengths of individual US techniques while mitigating their limitations. While standalone methods like B‐mode‐US, SWE, and CEUS provide valuable insights, their integration into an mpUS framework significantly enhances diagnostic precision. The adoption of mpUS in routine clinical practice can reduce diagnostic uncertainty, guide surgical planning, and inform decisions regarding malignancy and nerve‐sparing strategies. On the other hand, CCDS appears to have limited utility as a primary diagnostic tool but may still contribute useful information when used as part of a broader mpUS protocol. Overall, our results underscore the critical role of mpUS in advancing the preoperative assessment of patients with PGTs and supporting accurate and informed decision‐making in patient management.

In clinical practice, small, low‐grade PGTs with regular borders present a diagnostic and management challenge [60]. In our workflow, if such lesions display suspicious sonographic features—such as increased stiffness on SWE or hypoechoic areas in B‐mode US paired with abnormal enhancement in these areas on CEUS—we perform a USGB for further diagnostic clarification. However, some of these lesions may not exhibit any of the above‐mentioned signs of malignancy and therefore may not meet the criteria for USGB. In such cases, if malignancy is only identified postoperatively based on final histopathological analysis, the patient undergoes further staging and is presented to the interdisciplinary tumor board. A neck dissection, as outlined in our treatment protocol, is only performed if imaging reveals suspicious lymph nodes, in accordance with current guidelines and based on tumor board decision.

Upon histological confirmation of malignancy—whether pre‐ or postoperatively—the case is discussed in our interdisciplinary tumor board according to current guidelines (ASCO, ESMO and German AWMF guidelines [12, 13, 14]) to determine the optimal treatment strategy, balancing oncologic safety and functional preservation. If the tumor can be resected with clear margins without compromising the facial nerve, nerve preservation is prioritized. Nevertheless, patients are always informed preoperatively about the potential for facial nerve sacrifice and reconstruction options. This strategy ensures that appropriate oncologic treatment can still be initiated even when the diagnosis is established only after surgery and appears to be consistent with the recommendations of other working groups [13, 61].

One of the primary limitations of this study is the relatively small number of malignant PGTs included in the analysis. This limitation stems from the fact that malignant PGTs are rare, and their histological diversity further complicates obtaining a representative sample [13]. With numerous distinct types of malignancies affecting the parotid gland, it is challenging to generalize findings across the broad spectrum of tumor subtypes, which may affect the applicability of our results to all clinical scenarios. Although the overall sample size—particularly for malignant tumors—was limited, several measures were taken to enhance the statistical power of the study. These included a standardized imaging protocol using the same high‐end ultrasound device for all patients, a consensus‐based qualitative image interpretation by experienced readers, and a focused analysis of distinct tumor subtypes with clearly different sonographic features. Furthermore, strict inclusion criteria and the exclusion of technically insufficient image data minimized variability and increased internal validity. These methodological strategies contributed to strengthening the diagnostic conclusions despite the modest sample size.

Another challenge is that mpUS is not widely available in routine clinical settings. MpUS requires high‐end US machines equipped with advanced features such as SWE and CEUS. While SWE is now a standard feature in many newer devices, CEUS and full integration of mpUS modalities remain less accessible in many healthcare institutions due to equipment constraints, likely stemming from lower reimbursement rates compared to other imaging modalities [62, 63]. This limited accessibility may hinder the widespread adoption of mpUS as a routine diagnostic tool for PGTs, but its real‐time workflow may offset the costs associated with MRI in selected clinical settings.

A key limitation of this study is the exclusive use of qualitative assessment for interpreting CEUS and SWE data. This approach was intentional, as the study aimed to evaluate the clinical utility of real‐time, consensus‐based interpretation within routine diagnostic workflows, rather than to define or compare quantitative thresholds. Although quantitative SWE values have been explored in the literature, there are no universally accepted cut‐off values, primarily due to technical differences between ultrasound manufacturers, software algorithms, and measurement conventions [64, 65]. Many studies report stiffness in kilopascals (kPa), but these values are not directly measured. Instead, they are derived from shear wave velocity using the equation *E* = 3*ρv* ^2^, where *E* is Young's modulus, *ρ* is the assumed tissue density (typically 1000 kg/m^3^), and *v* is the shear wave speed [66]. This equation is based on assumptions of isotropic, homogeneous, and incompressible tissue—conditions generally applicable to the liver, for which SWE was originally developed [67]. In superficial structures such as the parotid gland, these assumptions may not hold. The relatively shallow location of parotid lesions alters wave propagation characteristics and may affect both shear wave velocity and the derived kPa values [42]. Additionally, the way different devices implement the conversion formula can result in variability, making quantitative SWE data difficult to compare across platforms [68].

Given these limitations, we deliberately focused on the qualitative interpretation of SWE elastograms. In our view, assessing whether a lesion appears “soft” or “hard” relative to surrounding glandular tissue offers more practical and reproducible diagnostic value than relying on absolute stiffness measurements. We therefore recommend future research aimed at developing standardized criteria—both qualitative and quantitative—specifically for superficial soft tissue applications such as the parotid gland.

Similarly, CEUS interpretation in our study was based on visual evaluation of enhancement patterns. Although consensus reading by two experienced examiners helped reduce inter‐observer variability, we acknowledge the inherent subjectivity of this approach. Future investigations should incorporate standardized reading protocols and formal inter‐reader reliability assessments to improve consistency and repsssroducibility across centers.

Beyond the general constraints discussed above, several methodological factors may limit the broader applicability of our findings. First, while tumor size was documented for the quantitative analysis, we did not perform a subgroup analysis based on lesion dimensions. Size may influence visual interpretation, particularly in SWE and CEUS, where larger or cystic lesions could alter the imaging characteristics. Second, although overall diagnostic accuracy was high, false positives and false negatives did occur, and their potential clinical consequences were not systematically analyzed. Third, all examinations were performed and interpreted in a high‐expertise, single‐center setting using consensus reading. While this improves internal consistency, it introduces potential operator dependency and may reduce generalizability to settings with less experience or different workflows. Additionally, the relatively small number of malignant tumors (*n* = 24) reflects the true distribution of parotid gland tumors in clinical practice, but it limits the statistical power for subgroup comparisons and validation of findings across tumor types. Future multicenter studies with standardized acquisition and interpretation protocols are needed to confirm reproducibility and broader clinical utility.

Despite these limitations, the findings of our study underscore the potential of mpUS to improve diagnostic precision and guide clinical decision‐making, even if its implementation requires careful consideration of technical and logistic factors. Our results suggest that the combination of different sonographic techniques in mpUS provides superior diagnostic accuracy compared to each modality alone, underscoring the value of using a multiparametric approach in patients with PGTs, which is also in line with previous studies [37, 69]. Future research should validate these findings in larger study populations with more malignant cases and explore ways to make advanced US technologies more widely available.

In conclusion, this study demonstrates that multiparametric ultrasound (mpUS), when combining B‐mode, shear wave elastography, and contrast‐enhanced ultrasound, shows promise in improving the preoperative diagnostic accuracy of parotid gland tumors compared to single‐modality ultrasound techniques. While our proposed diagnostic pathway may assist in differentiating benign from malignant lesions and guiding surgical planning, these findings should be interpreted cautiously. This was a single‐center study with a limited number of malignant cases, and the imaging interpretation was qualitative and consensus‐based. Furthermore, broader clinical adoption of mpUS may be limited by equipment availability, CEUS approval status in some regions, and the need for examiner training. Therefore, mpUS should not yet be considered a standalone diagnostic standard. Future multicenter studies are warranted to validate our findings, assess inter‐reader reliability, and evaluate the clinical impact and cost‐effectiveness of mpUS‐guided decision‐making in diverse healthcare settings.

## Conflicts of Interest

The authors declare no conflicts of interest.

## Data Availability

The data that support the findings of this study are available from the corresponding author upon reasonable request.
